# Evaluating Organisational Readiness for Advanced Access Implementation in Primary Healthcare: A Qualitative Study

**DOI:** 10.1111/jep.70714

**Published:** 2026-11-03

**Authors:** Gabriel Antônio Flores Chies, Gisele Teixeira de Souza, Maria Eugênia Bresolin Pinto, Alisia Helena Weis

**Affiliations:** ^1^ Postgraduate Program in Family Health Federal University of Health Sciences of Porto Alegre (UFCSPA) Porto Alegre Rio Grande do Sul Brazil; ^2^ Undergraduate Nursing Program Federal University of Health Sciences of Porto Alegre (UFCSPA) Porto Alegre Rio Grande do Sul Brazil; ^3^ Department of Public Health Federal University of Health Sciences of Porto Alegre (UFCSPA) Porto Alegre Rio Grande do Sul Brazil; ^4^ Department of Nursing Federal University of Health Sciences of Porto Alegre (UFCSPA) Porto Alegre Rio Grande do Sul Brazil

**Keywords:** appointments and schedules, health services accessibility, health services research, organisational innovation, practice evaluation, primary healthcare, qualitative research

## Abstract

**Rationale:**

Advanced access has been proposed to improve responsiveness in primary healthcare, but its sustainability depends on organisational conditions that are often insufficiently evaluated in territorially based public health systems.

**Aims and Objectives:**

To evaluate multiprofessional perspectives on organisational readiness and implementation factors influencing the transition from traditional appointment scheduling to advanced access in primary healthcare.

**Methods:**

A qualitative exploratory‐descriptive study with an interpretive perspective was conducted with 20 professionals working in Family Health Strategy teams in southern Brazil. Semi‐structured interviews were transcribed verbatim and analysed using thematic content analysis. Analysis was guided deductively by the organisational readiness for change framework and complemented by inductive coding.

**Results:**

Six evaluation domains were identified: management and leadership; human resources and infrastructure; work processes; culture and internal relationships; communication and community; and workload and well‐being. Leadership support, territorial sizing, workforce stability, interprofessional coordination, qualified initial reception and communication with the community shaped implementation. Participants perceived advanced access as capable of improving access and reducing conflict, but warned that insufficient planning, fragile institutional support and workload intensification could compromise sustainability.

**Conclusion:**

Advanced access should be evaluated as an organisational change rather than a technical adjustment to appointment books. Sustainable implementation requires readiness across governance, workforce, work processes, relational culture and community communication.

## 1 Introduction

Access is a core attribute of primary healthcare and a persistent challenge for health systems that combine high demand with limited workforce and infrastructure [1, 2]. In this context, appointment systems are not merely administrative devices: they shape how demand is recognised, prioritised and converted into care [3, 4, 5]. Traditional scheduling models can generate long waiting times, accumulated backlog, loss of responsiveness and conflict at the front door of services [4, 5, 6].

Advanced access was developed to reduce delays by balancing supply and demand, eliminating backlog and offering timely appointments according to clinical need and patient preference [7, 8]. Evidence suggests that advanced access may reduce waiting times and absenteeism, but its effects are inconsistent when the organisational conditions required for implementation are absent or unstable [9, 10, 11, 12, 13, 14, 15]. The model therefore demands more than a change in appointment availability [13, 14, 15]. It requires redesigned workflows, active demand management, interprofessional collaboration, information systems and a culture oriented to timely and comprehensive care [9, 10, 11].

In Brazil, the Family Health Strategy is organised territorially, with multiprofessional teams responsible for defined populations [3, 6]. This structure creates opportunities for continuity and community orientation, but it also makes access reforms vulnerable to population sizing, workforce turnover, municipal governance and local political pressures [3, 6, 16, 17]. International literature on advanced access remains strongly centred on physicians and nurses, whereas Brazilian primary care also relies on nursing technicians, dentists, psychologists and other professionals who participate in reception, demand management and care coordination [11, 12, 17].

Organisational readiness for change provides a useful evaluative lens for understanding implementation in this setting. Armenakis and Harris conceptualise readiness through dimensions such as discrepancy, appropriateness, efficacy, support and valence [18]. These dimensions help assess whether professionals perceive a problem requiring change, believe the proposed innovation is suitable, feel capable of implementing it, perceive institutional support and recognise valued benefits [18]. This study used this framework to evaluate the transition to advanced access in primary healthcare from a multiprofessional perspective.

The study aimed to evaluate professionals' perspectives on organisational factors influencing the transition from traditional scheduling to advanced access in Family Health Strategy services in southern Brazil. The research question was: what organisational, professional and contextual factors facilitate or hinder sustainable implementation of advanced access in primary healthcare?

## 2 Methods

### 2.1 Design

This was a qualitative exploratory‐descriptive study with an interpretive perspective. Reporting was guided by the Standards for Reporting Qualitative Research (SRQR) [19].

The design was appropriate because the objective was not to measure the effect of advanced access, but to evaluate how professionals interpreted and experienced the organisational conditions surrounding its implementation.

### 2.2 Setting and Participants

The study was conducted in Sapucaia do Sul, a municipality in the metropolitan region of Porto Alegre, southern Brazil. The municipality has progressively expanded primary healthcare since the 1990s and had substantial Family Health Strategy coverage [3, 20]. Sapucaia do Sul was selected because of its experience implementing advanced access, the researchers' connection with the municipal primary care network and the feasibility of access to professionals. Participants described 15 implementation or maintenance experiences involving 13 of the municipality's Family Health Strategy teams. Some teams had more than one experience, and some participants had been involved in multiple experiences. The reported experiences occurred between 2018 and 2024 and included full, partial and discontinued implementation. Organisational configurations included single and double teams, with reported population sizes ranging from approximately 2000−4000 people. These experiences were not assessed at a standardised point in the implementation process.

Participants were recruited by convenience for operational feasibility; the professional distribution reflected availability rather than predefined quotas. G.A.F.C. invited eligible professionals by email and telephone. Eligibility requires experience in the transition to or maintenance of advanced access, either in frontline care or in management. Direct experience meant involvement while working in clinical care in the teams; indirect experience meant involvement through management responsibilities. Some participants had experience in both roles. Professionals on leave during data collection were excluded. Twenty‐two professionals were invited; 20 were interviewed, and the remaining two were not interviewed because data collection had ended after informational adequacy was judged sufficient. The sample comprised physicians, nurses, dentists, nursing technicians and psychologists.

### 2.3 Data Collection

Recruitment and interviews took place between July and August 2025. Interviews were conducted individually and face to face by G.A.F.C. during reserved appointments at participants' workplaces, without interruptions and lasted approximately 30 min. G.A.F.C. was a family and community physician undertaking a master's degree in Family Health. He had worked for 5 years as a Family Health Strategy physician in the municipality and had received training in interviewing. Some participants were known to him, including former colleagues, while others were not; there was no hierarchical relationship between the interviewer and participants. Participants were informed that the interview formed part of his master's dissertation. The interview guide included sociodemographic and professional questions and five topic areas: the organisational system; key actors and leadership; change management processes; individual factors; and experiences with appointment scheduling models. The first four areas were informed by Amarantou et al. [21], and the last used a SWOT structure adapted from Lameirão et al. to explore advanced access [22]. Sociodemographic and professional questions addressed age, sex, race/colour, marital status, professional category, training and specialist qualifications, employment arrangements, working hours, other jobs, length of service and clinical or management roles at the time of interview and during the scheduling transition. The guide was not pilot tested or modified during data collection. Interviews were audio‐recorded, and field notes were made to complement contextual interpretation. No repeat interviews were conducted. Preliminary coding occurred during data collection. Recruitment was discontinued after three consecutive interviews generated no new codes, resulting in 20 completed interviews. Informational adequacy was assessed across the data set as a whole, not separately by profession or team. This stopping criterion supported the analysis of shared organisational factors but did not establish saturation within each professional group.

### 2.4 Data Analysis

Thematic content analysis followed Bardin's stages of pre‐analysis, material exploration, treatment and interpretation [23].

Analysis combined deductive and inductive procedures in a defined sequence. First, interview material was coded against the five organisational readiness dimensions—discrepancy, appropriateness, efficacy, support and valence—using specific operational definitions [18]. Second, each coded segment was assigned a polarity: positive if it favoured change or advanced access; neutral if its effect depended on context; and negative if it hindered change or advanced access. Third, secondary content coding following Bardin identified 55 emergent contents, which were refined into 30 subcategories and grouped into six empirical domains [23]. The five areas of the interview guide structured data collection, whereas the six empirical domains resulted from the analysis; they should not be interpreted as equivalent classifications. G.A.F.C. and G.T.S. independently read and coded the material. Segments with discrepant interpretations were reviewed by M.E.B.P. and A.H.W., and disagreements were resolved through consensus. The empirical domains were interpreted alongside the readiness dimensions and polarity assignments retained from the primary coding. The domains, therefore, identify the organisational areas in which implementation factors were expressed, while readiness dimensions describe their meaning for organisational change, and polarity describes whether they favoured or hindered implementation. No systematic comparisons between professions or teams were undertaken.

### 2.5 Ethical Considerations and Rigour

The study was approved by the Research Ethics Committee of the Federal University of Health Sciences of Porto Alegre before data collection (CAAE: 90087525.0.0000.5345). All participants provided written informed consent covering interviews, audio recording and publication of anonymised excerpts. G.A.F.C. transcribed the interviews and checked each transcript against the audio recording twice. Transcripts were returned to participants for review; no corrections or additions were made. Data were organised in Microsoft Excel spreadsheets using participant codes, with access restricted to the researchers involved in analysis. Credibility was supported by independent reading and coding by G.A.F.C. and G.T.S., review of disagreements by M.E.B.P. and A.H.W., consensus discussion and use of field notes to complement interpretation. G.A.F.C.'s familiarity with the local network facilitated access and contextual understanding but could also influence interpretation, as could prior working relationships with some participants. Independent coding and review provided opportunities to question these interpretations. Preliminary findings were not returned to participants for validation; transcript review was distinct from checking the analytic findings. No formal audit trail or external review was undertaken.

## 3 Results

### 3.1 Participant Characteristics

The study included 20 participants from different professional groups: physicians (25%), nurses (25%), dentists (25%), nursing technicians (15%) and psychologists (10%). Most participants were female (65%). Mean age was 42.3 years (SD 6.0), mean time since professional qualification was 16.7 years (SD 6.9) and mean length of service in the municipality was 10.5 years (SD 6.8). Ninety‐five percent worked 40 h or more per week in primary healthcare. Most participants (85%) had contact with the scheduling transition while working in Family Health Strategy teams, and some had also experienced it from municipal management positions (30%). These experiences involved 13 different teams and 15 implementation or maintenance experiences. The percentages for clinical and management experience used all 20 participants as the denominator and were not mutually exclusive.

### 3.2 Evaluation Domains

The analysis identified six domains that shaped implementation: management and leadership; human resources and infrastructure; work processes; culture and internal relationships; communication and community; and workload and well‐being. These domains operated across the readiness‐for‐change dimensions, showing that advanced access was experienced as a complex organisational intervention rather than a discrete scheduling technique (Figure 1 and Table 1). The analytical sequence can be illustrated by an excerpt from Nurse01: ‘Advanced access was already getting underway, but it was still something I had never worked with; I had not had that experience. I knew a little about it in theory and agreed with the system’. In the recorded analysis, the excerpt was primarily coded as appropriateness, assigned neutral polarity and secondarily coded under the emergent subcategory of knowledge within the human resources domain, presented here as human resources and infrastructure. The coding rationale was the participant's theoretical knowledge of and agreement with advanced access, expressed alongside a lack of practical experience. This illustrates how a segment retained its readiness dimension and polarity while contributing to an empirically derived subcategory and domain.

**Figure 1:**
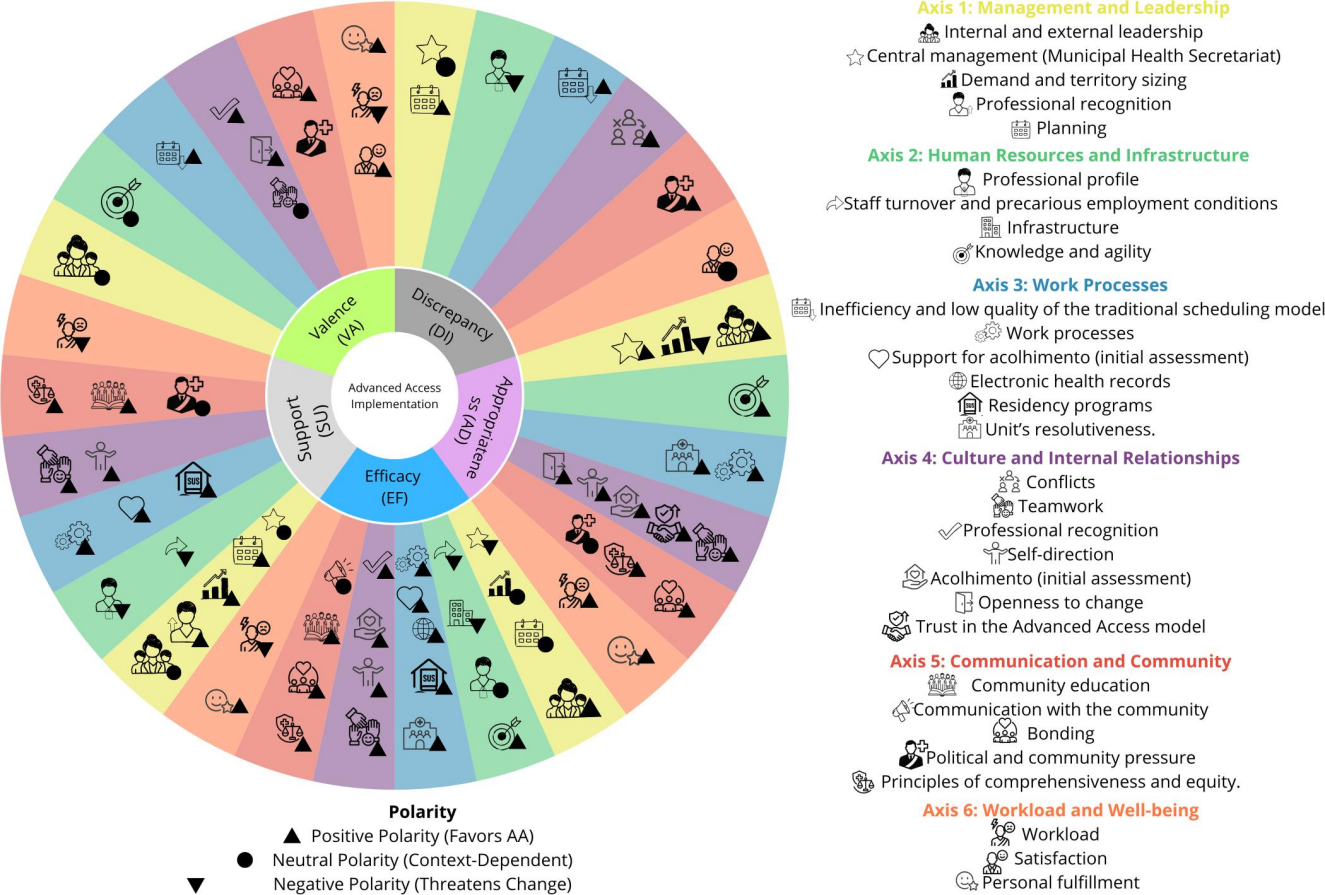
Six empirical domains and their subcategories interpreted through the five dimensions of organisational readiness for change. The domains identify organisational areas of implementation; the readiness dimensions provide the theoretical interpretation. Symbols indicate whether the reported factor favours change, depends on context or threatens change. The analysis first assigned readiness dimensions and polarity, then grouped 55 emergent contents into 30 subcategories and six domains. A recorded example linking theoretical knowledge to appropriateness, neutral polarity and the human resources domain is provided in Section 3.2.

**Table 1:** Evaluation domains and implications for implementation.

Domain	Main evaluation finding	Readiness dimension most affected
Management and leadership	Local leadership enabled implementation, but absence of municipal direction produced heterogeneity across units.	Support; efficacy
Human resources and infrastructure	Professional profile, stability, training, infrastructure and electronic records influenced capacity to implement the model.	Efficacy; support
Work processes	Qualified initial reception, shared schedules and role clarity determined whether demand could be managed safely.	Appropriateness; efficacy
Culture and relationships	Trust, collaboration and openness to change reduced resistance and supported continuity.	Efficacy; valence
Communication and community	Community understanding, political pressure and bonding influenced acceptance of the model.	Appropriateness; support
Workload and well‐being	Implementation increased work intensity but could improve satisfaction when conflicts decreased and resolutiveness improved.	Valence

### 3.3 Management and Leadership

Leadership emerged as the most visible implementation condition. Some teams were able to begin or sustain advanced access because local leaders mobilised professionals, organised planning meetings and defended the change when resistance appeared. However, participants also described the absence of clear central management directives as a source of instability. Autonomy allowed local experimentation, but it also produced heterogeneous access rules across units and confusion for users.

Sapucaia do Sul does not have uniformity [in scheduling]… it is quite confusing for users.

(Nurse05)

Territorial and demand sizing were closely linked to leadership and planning. Participants described teams with smaller or better‐sized populations as more able to sustain the model. In contrast, poorly estimated populations and suppressed demand led to renewed backlog, schedule congestion and deterioration of access. Professional recognition by management was also described as a condition for sustaining the effort required by the new model.

### 3.4 Human Resources and Infrastructure

Implementation depended on professional profile, workforce stability, knowledge of primary care and availability of minimum infrastructure. Participants valued agility, flexibility, resolutiveness and willingness to discuss cases. The role of nurses and nursing technicians was especially relevant because initial reception was central to demand management. At the same time, staff turnover and precarious contracts weakened implementation because teams had to repeatedly rebuild shared routines and train new professionals.

For Advanced Access to function, professionals needed preparation… One person changes in that team and it already puts everything at risk.

(Nurse05)

Infrastructure also shaped efficacy. Inadequate space for reception or consultation limited quality and increased tension. Electronic health records, when available and integrated, were described as facilitators because they allowed real‐time visibility of the unit's demand and supported coordinated decisions.

### 3.5 Work Processes

Work processes determined whether advanced access was interpreted as comprehensive access or merely as walk‐in care. Participants emphasised the need for clear flows, shared schedules, team‐wide responsibility and permanent education. Reception was not understood as a simple triage point, but as a qualified organisational and care practice based on listening, risk assessment, problem solving and access to appropriate professionals.

The schedule belongs to the team, not just to the physician. If each professional establishes their own way of working, it becomes chaotic.

(Nurse03)

The evaluation showed that advanced access can be distorted when implementation is reduced to increasing the number of consultations or shortening consultation time. Professionals warned that access reform must preserve continuity, quality and comprehensiveness. The strongest work‐process facilitators were shared decision‐making, support from higher‐level professionals to reception staff, case discussion and interconsultation.

### 3.6 Culture and Internal Relationships

Team culture influenced how the change was understood and adopted. Cohesive teams with trust, dialogue and a user‐centred orientation had fewer barriers. In these settings, professionals interpreted access reform as a shared responsibility and not as an individual burden. Conversely, unresolved conflicts, rigid professional boundaries and resistance to leaving established routines reduced collective efficacy.

It has to be a team that works cohesively… the interaction between reception and the physician is the most important part for spontaneous demand to function.

(NursingTech03)

Participants also associated openness to change with humility and critical reflection on work processes. This suggests that readiness was not only technical but relational and cultural. Teams that questioned their own routines were more able to adapt the model to local needs while preserving its central principles.

### 3.7 Communication and Community

Advanced access changed the relationship between services and the community. Participants reported initial resistance from users accustomed to traditional scheduled appointments. Lack of explanation generated insecurity, complaints and political pressure. Community education was therefore an implementation condition, not a peripheral activity. Professionals needed to explain why some users would be seen earlier, how reception worked and how the new model differed from queue distribution.

We need to build this culture within the population, which is more complex and takes time.

(Physician05)

When communication improved, and the model became familiar, participants described increased bonding with the multiprofessional team, reduced conflict at reception and greater perception that the unit remained open to the community. The model also helped decentralise care from the physician by increasing contact with nurses, technicians, dentists and other team members.

### 3.8 Workload and Well‐Being

Workload was the main ambivalent consequence of implementation. Many participants stated that advanced access made professionals work more, especially those responsible for reception and those committed to solving problems. The model revealed suppressed demand and increased the flow of people through the unit. Without adequate staffing, territorial sizing and role distribution, this increased work intensity could generate exhaustion.

It takes more work to listen to people, it takes more work for everyone; the team has to be willing.

(Nurse01)

At the same time, several participants described greater satisfaction, professional fulfilment and reduced conflict when the model was well implemented. The perceived value of the change was linked to resolutiveness, stronger bonds and the possibility of going home with fewer unresolved access problems. Thus, valence depended on whether increased effort was matched by visible improvements in care and team functioning.

## 4 Discussion

This qualitative evaluation indicates that advanced access implementation in territorially based primary healthcare depends on organisational readiness across multiple levels. The findings extend previous literature by showing how readiness is constructed through the interaction of leadership, workforce capacity, work‐process design, relational culture, community communication and workload. The multiprofessional composition of the sample is particularly important because the daily organisation of access in Brazilian primary care is not restricted to physicians or nurses.

The management and leadership domain was central to the support dimension of readiness. International studies on advanced access emphasise the need to balance supply and demand and to maintain consistent improvement work [7, 11, 12, 15, 24]. In this study, local leadership enabled teams to experiment, but the absence of municipal governance produced heterogeneous rules and user confusion. This finding suggests that decentralised innovation requires a minimum degree of system‐level coordination. Without common principles, monitoring and support, advanced access risks becoming a set of disconnected local arrangements rather than a coherent access policy.

Human resources and infrastructure shaped perceived efficacy. The model increased interdependence among professionals: reception staff needed nurse and physician support; physicians depended on qualified reception and shared triage; dentists and psychologists were affected by changes in demand flow. Workforce instability therefore threatened implementation because new staff could disrupt the relational and procedural agreements that made the model function. This finding is consistent with implementation theory, which treats organisational capacity and local resources as conditions for routinisation [9, 18, 25].

Work processes were central to the evaluation because they mediated the relationship between the model and everyday care. Advanced access was sustainable when translated into clear routines for reception, agenda management, case discussion and interprofessional support. It was fragile when interpreted only as same‐day availability or as pressure to increase consultation numbers. This distinction matters for clinical practice: an access reform that reduces waiting time but undermines continuity or comprehensiveness may fail to improve care quality [8, 9, 13].

Culture and internal relationships influenced efficacy and valence. The literature on organisational culture in healthcare suggests that innovations spread more easily when they align with shared values and cooperative work [26, 27, 28]. The present study supports this view. Teams with trust and communication perceived advanced access as a collective project. Teams marked by resistance or rigid professional boundaries experienced the same model as a threat or additional workload.

Communication with the community emerged as an evaluative criterion for implementation. In public primary care systems, access models are experienced not only by professionals but also by users who hold expectations about queues, order of arrival and scheduled appointments [13]. The findings suggest that community education should be included in implementation plans and evaluation frameworks. Without it, political pressure and complaints may destabilise changes even when internal team processes are improving [16].

Finally, workload and well‐being revealed the central tension in advanced access. Opening the system to suppressed demand may initially increase work intensity [9]. However, participants also reported reduced conflict, greater satisfaction and stronger bonding when access improved. The evaluative question is therefore not whether advanced access increases or decreases work in the short term, but whether the organisation creates conditions for increased responsiveness without transferring unsustainable burden to frontline teams. Improved responsiveness may therefore coexist with greater work intensity: opening access can make previously unmet needs visible without, by itself, expanding the resources available to address them. In the participants' accounts, the tension was particularly evident when staffing, territorial sizing and role distribution were inadequate. The sustainability of advanced access should therefore be considered not only in terms of staff well‐being, but also in relation to the potential consequences of workload and staffing pressures on the completeness of care delivered [29]. Evidence from Italian home nursing care links perceived workload and staffing pressures to missed care, supporting attention to this possibility while recognising differences between home nursing and Brazilian multiprofessional primary care [29]. The present study did not measure missed care or establish that advanced access caused omissions.

Taken together, the findings suggest that improved access becomes an unsustainable transfer of burden when newly visible demand is accommodated through greater individual effort rather than adjustments to team capacity and work organisation. Understaffing, workforce turnover, unclear responsibilities and weak managerial support may leave frontline professionals to absorb the gap between demand and capacity. Local autonomy and professional commitment can facilitate implementation, but cannot substitute indefinitely for adequate resources and institutional support. Conversely, shared reception responsibilities, interprofessional backup, realistic population sizing and ongoing review of demand and workload may help teams preserve responsiveness and comprehensiveness. These are organisational implications of the qualitative findings rather than tested causal effects.

### 4.1 Implications for Evaluation and Clinical Practice

The findings suggest that evaluations of advanced access should not rely solely on indicators such as waiting time, absenteeism or number of consultations. These indicators are important but insufficient. Improved access describes how readily patients enter the service, whereas activity counts describe the volume of contacts; neither, on its own, captures the complexity of care required after entry. Similar consultation volumes may involve substantially different needs for assessment, counselling, coordination and follow‐up. Evaluations should therefore consider care complexity alongside access and activity when assessing workload, workforce requirements and sustainability. Recent nursing literature supports making care needs more visible through structured clinical documentation, although measures developed in hospital settings require adaptation and validation before use in primary care [30, 31, 32]. A broader evaluation should examine organisational readiness, population sizing, workforce stability, reception quality, interprofessional coordination, community understanding and professional well‐being. In practice, managers should treat advanced access as a service redesign process requiring preparation, training, monitoring and adaptive support (Table 2).

**Table 2:** Practical evaluation questions for advanced access implementation.

Evaluation area	Questions for managers and evaluators
Governance	Are there clear municipal or organisational principles for the model, while allowing local adaptation?
Demand and capacity	Has the team assessed population size, suppressed demand and appointment capacity before implementation?
Workforce	Are staffing, training and employment stability sufficient to sustain the new workflow?
Work processes	Are reception, risk assessment, interprofessional support and agenda rules clearly defined?
Community	Do users understand how the new access pathway works and why priorities may differ from order of arrival?
Well‐being	Is workload being monitored alongside access indicators and user satisfaction?

### 4.2 Strengths and Limitations

The study has limitations. It was conducted in one municipality and used a convenience sample, which may limit transferability. The data reflect professionals' perspectives and do not include direct accounts from users or central policymakers. The presence of researchers with experience in the local primary care context may have facilitated access and trust but also required reflexive attention to possible interpretive influence. Data collection occurred during a specific period and may not capture later changes in municipal management or service organisation. Nevertheless, the study provides a detailed multiprofessional evaluation of a complex access reform and includes professional categories often underrepresented in the advanced access literature. The experiences described were retrospective, spanning 2018−2024 and differed in implementation maturity; recall and temporal heterogeneity may therefore affect interpretation. Informational adequacy was evaluated across the data set, and the small numbers of nursing technicians (*n* = 3) and psychologists (*n* = 2) limit profession‐specific interpretations. No systematic comparisons between professions or teams were undertaken. Although transcripts were returned for review, the findings themselves were not validated by participants, and no formal audit trail or external analytic review was undertaken.

### 4.3 Future Research

Future studies should incorporate user perspectives, compare municipalities with different levels of institutionalisation of advanced access and examine longitudinal sustainability. Mixed‐methods studies could combine qualitative readiness evaluation with indicators of waiting time, continuity, absenteeism, workload and user experience. Future evaluations should complement access and activity indicators with measures capable of capturing patient care complexity to better understand the actual care and resource demands associated with changes in access [29, 30, 31]. Suitable measures should be selected or developed for the multiprofessional and longitudinal nature of primary care, rather than transferred directly from inpatient settings. Studies should also examine whether changes in access are accompanied by unmet care needs or delayed essential activities, alongside workforce capacity and professional well‐being [32]. The findings also support the development of instruments to assess organisational readiness for access reforms in primary healthcare.

## 5 Conclusion

Advanced access implementation in primary healthcare is a complex organisational change that cannot be adequately evaluated as a simple alteration of appointment scheduling. In this study, sustainability depended on leadership, workforce capacity, infrastructure, clear work processes, collaborative culture, communication with the community and attention to workload. Organisational readiness for change provided a useful framework for interpreting these conditions and identifying where implementation may succeed or fail. For managers and evaluators, the central implication is that access reform requires coordinated preparation, ongoing support and assessment of both operational outcomes and the lived experience of frontline teams.

## Author Contributions

**Gabriel Antônio Flores Chies:** conceptualisation, methodology, investigation, formal analysis, visualisation, writing – original draft, writing – review and editing, project administration. **Gisele Teixeira de Souza:** formal analysis, visualisation, writing – original draft, writing – review and editing. **Maria Eugênia Bresolin Pinto:** and **Alisia Helena Weis:** conceptualisation, methodology, formal analysis, writing – review and editing, supervision.

## Funding

The authors have nothing to report.

## Ethics Statement

The study was approved by the Research Ethics Committee of the Federal University of Health Sciences of Porto Alegre (CAAE: 90087525.0.0000.5345). All participants provided written informed consent.

## Conflicts of Interest

The authors declare no conflicts of interest.

## AI Use Statement

ChatGPT 6.1 Sol. (OpenAI) was used to support translation of interview excerpts and academic language refinement, and to assist in drafting proposed manuscript revisions. The fidelity of translated interview excerpts to the Portuguese originals was checked by two researchers. AI did not generate interview data or perform the empirical coding. The researchers retained responsibility for study design, analysis, interpretation and the manuscript's substantive claims.

## Data Availability

The qualitative data generated and analysed during the current study are not publicly available because they contain interview material that could compromise participant confidentiality. Anonymised excerpts supporting the findings are included in the manuscript. Additional information may be available from the corresponding author upon reasonable request and subject to ethical approval.
